# Progress of bone marrow mesenchymal stem cell transplantation on neural plasticity in brain

**DOI:** 10.3389/fcell.2025.1589169

**Published:** 2025-06-10

**Authors:** Wenjing Li, Xiaohua Liu, Jingjing Li

**Affiliations:** ^1^ Physical Education College, Shanghai University, Shanghai, China; ^2^ School of Sport, Exercise and Health Sciences, Loughborough University, Loughborough, United Kingdom; ^3^ School of Exercise and Health, Shanghai University of Sport, Shanghai, China

**Keywords:** bmscs, neural plasticity, neurogenesis, inflammation, oxidative stress, exosomes

## Abstract

Stem cells are cells with strong proliferation and differentiation abilities. Among all stem cells, bone marrow mesenchymal stem cells (BMSCs) have been extensively studied. BMSCs have the ability to self-renew and differentiate into nerve cells, and participate in cell migration and survival. These cells can also secrete neurotrophic factors through paracrine pathways to affect neural plasticity. Transplantation of BMSCs can affect neural plasticity and is the main treatment method for stroke or other traumatic brain diseases. This article elaborates on the role of BMSC transplantation in neural plasticity, neurotrophic factors, and synaptic changes, and comprehensively analyzes its potential molecular mechanisms to provide a theoretical basis for clinical treatment of brain diseases.

## 1 Introduction

Bone marrow mesenchymal stem cells (BMSCs) originate from the mesoderm during early embryonic development and subsequently migrate to the bone marrow cavity, forming a stem cell niche within the marrow stroma. BMSCs are characterized by the expression of typical surface markers, including CD73, CD90, and CD105, while lacking hematopoietic lineage markers such as CD34, CD45, and CD11b. BMSCs are a population of adult stem cells with biological properties, multipotent differentiation potential, and self-renewal capacity. Under specific conditions, they can differentiate not only into mesoderm-derived lineage cells—such as osteocytes, chondrocytes, and cardiomyocytes—but also undergo transdifferentiation across germ layers, giving rise to neurons, glial-like cells, and hepatocytes (Chen et al., 2016). Furthermore, most BMSCs reside in the G0/G1 phase of the cell cycle, confirming their robust self-renewal ability. Due to their ease of isolation from adult tissues, extensive proliferative capacity, and ability to differentiate into various cell lineages, BMSCs are regarded as one of the most extensively studied regenerative cell types in current research (Liu J. et al., 2022). As a core component of the bone marrow microenvironment, BMSCs provide mechanical support *in vivo*, not only offering structural assistance to hematopoietic stem cells but also maintaining the physical stability of the marrow cavity. Moreover, BMSCs exhibit immunomodulatory functions and paracrine activity, enabling them to migrate to sites of tissue injury. Under the induction of specific factors, they can differentiate into specialized cell types or secrete relevant cytokines, thereby contributing to tissue repair and regeneration (Mohanty et al., 2020). Given their multilineage differentiation potential, self-renewal capacity, and immunoregulatory properties, BMSCs have emerged as a critical cell source for gene therapy, tissue engineering, and regenerative medicine.

Neuroplasticity, defined as the ability of the nervous system to reorganize its structure or function in response to environmental changes, represents a major focus of current research. Studies have demonstrated that interventions such as exercise, nutritional supplementation, rehabilitation training, and stem cell therapy can confer benefits to overall health and cognitive function by promoting neuroplasticity (Zhao et al., 2020; Trinchese et al., 2024; Huang et al., 2023). Among these approaches, stem cell therapy has emerged as a particularly promising strategy due to its capacity to facilitate neuroregeneration, with key cell types including embryonic stem cells (ESCs), neural stem cells (NSCs), mesenchymal stem cells (MSCs), and bone marrow mesenchymal stem cells (BMSCs) (Chrostek et al., 2019). BMSCs, in particular, are regarded as a prospective cellular therapy for neural injuries owing to their accessibility, multilineage differentiation potential, self-renewal capacity, neurotrophic properties, and immunomodulatory effects. Accumulating evidence suggests that BMSC transplantation may exert therapeutic effects on neurological disorders by modulating neuroplasticity; however, the underlying molecular mechanisms remain incompletely elucidated, necessitating further investigation to advance clinical translation (Chrostek et al., 2019). Therefore, this review synthesizes existing evidence on the impact of BMSC transplantation on neuroplasticity, aiming to clarify the associated molecular mechanisms and provide a theoretical foundation for future clinical applications in the treatment of neurological diseases.

## 2 Effect of BMSCs transplantation on neural plasticity

Neuroplasticity includes neural tissue plasticity, neuronal or glial plasticity, and synaptic plasticity. In general, the brain can exhibit plasticity. when physiological needs arises, neural activity changes, or neural tissues are damaged (Cohen et al., 2017). Studies have shown that BMSCs transplantation plays an important role in the repair of nervous system, and its mechanism involves multi-channel regulation of neural plasticity. This part systematically reviews how BMSCs can improve neural function by promoting neurogenesis, regulating the secretion of neurotrophic factors and enhancing synaptic plasticity.

### 2.1 Impact of BMSC transplantation on neurogenesis

Neurogenesis is a complex process, in which neural stem cells and neural progenitor cells (NPCs) undergo division and differentiation, ultimately producing fully functional neurons. It mainly includes stages, such as cell proliferation, migration, differentiation, and survival, and plays a key role in neural plasticity, brain homeostasis, and maintenance of the central nervous system (Ribeiro and Xapelli, 2021). Adult neurogenesis mainly occurs in two main regions of the brain: the dentate gyrus of the hippocampus and the subventricular zone (SVZ) (Cope and Gould, 2019). This process is influenced by various factors, including exercise, diet, environment, and aging (Poulose et al., 2017), which can regulate neurogenesis and affect the production of new neurons in the adult brain.

Research has demonstrated that BMSC transplantation exhibits considerable potential for treating neurological disorders, with one of its core mechanisms involving the enhancement of neuroplasticity through promoting endogenous neurogenesis (Shiota et al., 2018). BMSC transplantation can influence neurogenesis through both direct and indirect pathways: directly by migrating and differentiating into functional neurons or glial cells to replace damaged neural cells or through cell-cell contact to regulate neurogenesis, and indirectly via paracrine effects and immunomodulation. In male rat middle cerebral artery occlusion (MCAO) models, intracarotid injection of 2 × 10^6^ BMSCs increased the numbers of 5-bromo-2′-deoxyuridine (BrdU)- and Ki67-positive cells in the SVZ. Furthermore, BMSC transplantation promoted the differentiation of proliferating cells into astrocytes and upregulated the expression of bone morphogenetic proteins two and 4, gap junction protein connexin-43, and synaptophysin in the ischemic border zone, thereby facilitating functional recovery after stroke (Zhang et al., 2006). Shen et al. (Shen et al., 2006) showed that intracarotid injection of 2 × 10^6^ BMSCs increased both the quantity and density of Ki-67-positive proliferating cells and NG2-positive cells (a specific marker of oligodendrocyte progenitor cells) in the corpus callosum of MCAO rats, leading to improved neurological function. Another study reported that tail vein injection of 3 × 10^6^ BMSCs similarly increased BrdU-positive cells in the SVZ of female MCAO rats (Chen et al., 2003). Additionally, significant reductions in apoptotic cells and basic fibroblast growth factor (bFGF)-immunoreactive cells were observed in the ischemic border zone, accompanied by marked neurological improvement (Chen et al., 2003). These findings collectively indicate that BMSC transplantation can exert comparable neuroprotective effects on neurogenesis regardless of differences in sex, dosage, or transplantation route. Although all three studies observed BMSC-induced enhancement of neural proliferation, the effects may vary depending on administration methods and doses. For instance, intracarotid injection may more directly target ischemic areas, while tail vein injection requires cells to cross the blood-brain barrier. Moreover, the hormonal environment in female rats might influence the proliferative effects of BMSCs, necessitating further investigation into potential sex differences through controlled experiments to clarify these factors. Within 3 months post-transplantation, BMSCs actively proliferated toward lesions and partially expressed neuronal phenotypes in host brains (Yano et al., 2005). Notably, animal models receiving BMSC transplants exhibited significant improvements in both apoptosis reduction and neurological functional recovery, accompanied by enhanced cell proliferation and increased synaptophysin (SYP) expression, suggesting that BMSC transplantation may also influence neuroplasticity through additional pathways. Current evidence indicates that BMSC transplantation primarily regulates neuroplasticity through indirect mechanisms, though direct effects (such as local cell replacement or acute injury repair) may play crucial roles under specific conditions. However, further experiments are required to distinguish these mechanisms. Additionally, the optimal BMSC delivery method remains unconfirmed, and quantitative data are lacking regarding their differentiation ratios into functional neurons, oligodendrocytes, or astrocytes, as well as their survival rates and functional integration efficiency *in vivo*. Addressing these issues will be essential for maximizing the therapeutic efficacy of BMSC transplantation.

### 2.2 Effect of BMSCs transplantation on neurotrophic factor regulation

BMSCs possess not only multipotent differentiation potential but also secrete various bioactive molecules through paracrine mechanisms, including brain-derived neurotrophic factor (BDNF), vascular endothelial growth factor (VEGF), and nerve growth factor (NGF). These factors may play crucial roles in modulating neuroplasticity.

A study reported that rodents with MCAO exhibited beneficial effects within days after BMSC transplantation, while only a minimal fraction of BMSCs expressed neural markers. The complete differentiation of BMSCs into injured neural cells and their integration into complex neural networks typically requires weeks to months. This finding confirms that the protective effects of BMSCs cannot be attributed solely to newly generated neural cells. Studies have demonstrated that BMSCs can selectively migrate to sites of injured tissue, interact with brain cells, and subsequently stimulate the production of neurotrophic factors. This interaction with resident brain cells and the release of neurotrophic factors are considered key mechanisms underlying the therapeutic effects of BMSCs in promoting neural repair and recovery (Zhong et al., 2020). Liu et al. (Liu Y. et al., 2022) investigated the therapeutic efficacy of BMSC transplantation in ischemic stroke with comorbid hypertension using a male spontaneous hypertensive rat MCAO model. Intravenous injection of 1 × 10^6^ BMSCs resulted in significantly increased expression of VEGF and glial cell line-derived neurotrophic factor (GDNF) in the peri-infarct cortex at 3 days post-transplantation, suggesting BMSCs may induce an early neurotrophic response in the brain. Furthermore, the study observed progressive increases in VEGF and GDNF protein levels over time. Although temporal patterns of neurotrophic factor changes varied, the overall trend of increased expression remained consistent. Beyond VEGF and GDNF, other critical neurotrophic factors including insulin-like growth factor-1 (IGF-1) (Naal et al., 2017) and bFGF (Chen et al., 2003) also showed elevated levels following BMSC transplantation. Kim et al. (2017) demonstrated that intralesional BMSC transplantation in male rats with spinal cord injury significantly improved BBB scores while upregulating BDNF and synapsin-I expression. Subsequent work by the same group revealed elevated BDNF, TrkB, and ERK1/2 protein levels in BMSC-treated animals, suggesting BMSCs may promote neuroplasticity through BDNF-TrkB mediated ERK1/2 signaling activation (Kim et al., 2018). These findings were corroborated by another study showing BMSC transplantation in traumatic brain injury rats enhanced BDNF/TrkB expression and activated the ERK1/2 pathway (Shin et al., 2016). Guo et al. (2017) demonstrated that intravenous injection of BMSCs into TBI mice significantly upregulated the expression levels of VEGF and Ang-1 proteins in brain tissue. *In vitro* studies confirm that BMSCs can be induced to differentiate into neurotrophic factor-secreting cells, producing BDNF, GDNF, VEGF and NGF. One study reported 41.19% ± 13.24% of BMSCs showed positive BDNF expression (Sadan et al., 2008). Moreover, GDNF elevation following BMSC administration creates a favorable microenvironment for local cellular repair and migration of neuroblasts from the SVZ, contributing to functional improvement (Shen et al., 2010). This multifactorial neurotrophic capacity strongly supports the therapeutic potential of BMSCs for neurological disorders (Martínez-Morales et al., 2013). Collectively, these findings indicate BMSC transplantation induces coordinated changes in multiple neurotrophic factors, promoting neuroplasticity through multidimensional modulation of TrkB-BDNF signaling pathway. Future studies should focus on optimizing intervention strategies to maximize this therapeutic potential.

Neurotrophic factors also play a role in the differentiation of BMSCs. Researchers have studied the effect of GDNF on the differentiation of BMSCs. They found that GDNF induced BMSCs to differentiate into neuron-like cells and enhanced the expression levels of neuronal markers, including nestin and neuronal cell adhesion molecules (Ma et al., 2020). Neurotrophin-3 (NT-3) may affect the differentiation of BMSCs. Overexpression of NT-3 promotes the differentiation of BMSCs into neurons both *in vivo* and *in vitro*, while silencing NT-3 inhibits the differentiation of BMSCs (Yan et al., 2021). In addition, BDNF, as another neurotrophic factor, can promote the survival and differentiation of neuronal tissue and may participate in neuroprotective effects. According to reports, BDNF can improve the survival rate of neurons after 4 weeks of BMSC transplantation. However, when BDNF production is silenced in BMSCs through gene therapy, the cells cannot survive in the first week of transplantation and do not exert neuroprotective effects (Ritfel et al., 2015). This finding comfirms the neuroprotective role of BDNF during BMSC transplantation. Neurotrophic factors not only affect neural repair and recovery through their effects on brain cells, but also directly affect bone marrow stromal stem cells themselves, thereby promoting their differentiation into neural cell types. This dual action mechanism further increases the complexity of the therapeutic potential of bone marrow stromal stem cells and emphasizes their significance in promoting neural plasticity and functional recovery in neurological diseases.

In general, BMSC transplantation plays a crucial role in regulating neural plasticity through neurotrophic factors to exert its neuroprotective effects. Changes in neurotrophic factors induced by BMSCs may be due to several factors: the role of BMSCs as neurotrophic factor-secreting cells and the role of BMSCs in stimulating astrocytes to produce neurotrophic factors. Nevertheless, many questions should be considered: which factor plays a more important role among various neurotrophic factors, or which factor is a more effective therapeutic target. BMSCs are considered cells that secrete neurotrophic factors, but which factors cause their secretion remains unclear; as such, in-depth research should be conducted. Effectively addressing these issues may provide useful information for improving neural plasticity in BMSC transplantation.

### 2.3 Impact of BMSC transplantation on synaptic plasticity

The structure of synapses determines the directional transmission of neural signals and synaptic plasticity, serving as the foundation for learning, memory, and nervous system functional regulation. Synaptic plasticity refers to the characteristic or phenomenon where synapses can undergo relatively long-lasting changes in morphology and function (Magee and Grienberger, 2020). Studies have shown that significant changes occur in synaptic structure and function after brain injury (Gao et al., 2011). Shen et al. (2021) found that in male MCAO mouse models, dendritic spine density decreased, total dendritic branch length shortened, and synaptic marker proteins (Synapsin I, synaptophysin, and PSD95) were significantly reduced. Another study reached similar conclusions, showing that in male MCAO rat models, synaptic structure underwent severe alterations, manifested as perisynaptic edema, vacuolation, synaptic cleft widening, reduced synaptic vesicles, irregular morphology, and significantly decreased vesicle numbers (Lan et al., 2025). Research has found that BMSC transplantation not only promotes neurogenesis and secretes various neurotrophic factors, but also regulates synaptic plasticity through multiple mechanisms, including promoting synapse formation, enhancing synaptic transmission efficiency, and maintaining synaptic morphological and functional stability, thereby enhancing neuroplasticity. He et al. (2017) observed changes in neuroplasticity by injecting 1 × 10^6^ BMSCs into the carotid artery of male rats, and found significantly increased gene and protein expression levels of SYP and GAP43, improving synaptic plasticity and thereby promoting neuroplasticity. SYN is a key protein in synaptic signal transduction. Zhang et al. (2018) investigated the effects of intravenous, intra-arterial, and intracerebral BMSC injections on neuroplasticity, and found that all three transplantation methods significantly increased the number of SYN-positive cells, enhanced synapse formation, and promoted neurological functional recovery. Dendritic spines are small protrusions on neuronal dendrites that serve as the main postsynaptic sites for excitatory synapses, and their morphological and numerical changes directly reflect the state of synaptic plasticity. Xu et al. (2024) injected 1 × 10^6^ BMSCs into male MCAO mice via tail vein and found significant enhancement in various structural parameters of layer V pyramidal neuron dendrites, including total length, branch number, intersection number, and dendritic spine density. LTP is an important manifestation of synaptic plasticity, but there are few studies on how BMSC transplantation affects LTP.

In addition, BMSC transplantation has demonstrated beneficial effects on axonal plasticity. Studies have shown that BMSC treatment increases post-stroke axonal density and enhances both intercortical and intracortical axonal connections (Lin et al., 2021). Following MCAO, BMSC therapy promotes axonal growth in white matter, as evidenced by increased expression of NF-200 and GAP-43, along with elevated numbers of NF-200-positive fibers (Liu et al., 2010). Similarly, tail vein injection of BMSCs downregulates the expression of Nogo-A, an axonal extension inhibitory molecule, in MCAO rats, further confirming BMSCs’ ability to promote axonal regeneration and remodeling (Shen et al., 2008). Moreover, BMSC transplantation plays a crucial role in post-stroke axonal sprouting and remyelination. The observed increases in axonal sprouting and myelination around regenerating axons may underlie the therapeutic benefits of BMSCs (Shen et al., 2006). Research indicates that BMSC-derived exosomes (BMSCs-exos) significantly restore impaired basal synaptic transmission and synaptic plasticity while improving spatial learning and memory. These extracellular vesicles (EVs) also markedly suppress ischemia-induced pathogenic expression of COX-2 in the hippocampus. The improvement in synaptic function following transient global ischemia may be partially attributed to this inhibition of pathogenic COX-2 expression (Deng et al., 2017).

**FIGURE 1:**
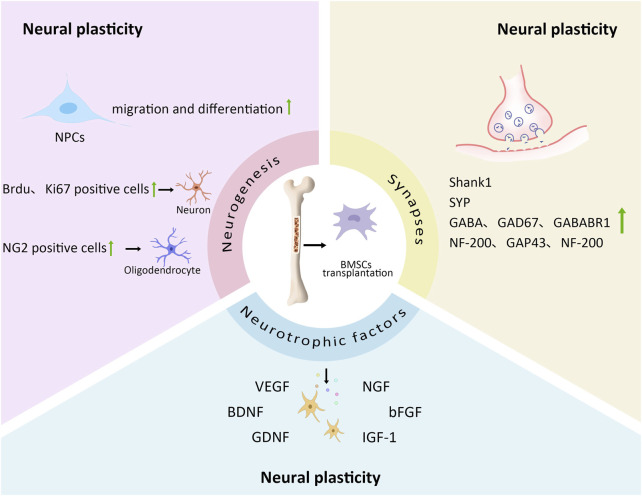
The effect of BMSCs transplantation on neural plasticity. BMSCs transplantation can play a role in neural plasticity by affecting neurogenesis, neurotrophic factors and synapses. In neurogenesis, BMSCs transplantation promotes the migration and differentiation of NPCs; The number of Brdu and Ki67 positive cells in SVZ is increased; The number and density of Ki67 and NG2 positive cells in corpus callosum is increased. In terms of neurotrophic factors, BMSCs transplantation releases a large number of VEGF, BDNF, GDNF, NGF, bFGF, IGF-1. They can improve neural plasticity. In terms of synapse, BMSCs transplantation increases the expression of synapse related factors such as Shank1, SYP, GABA, GAD67, GABABR1, NF-200, GAP43, which promotes axon regeneration and axon remodeling.

In summary, across various rodent models of stroke and neurodegenerative disorders (including MCAO and AD), BMSC transplantation has consistently demonstrated positive effects by: promoting neurogenesis, enhancing neurotrophic factor secretion, improving synaptic structure, increasing neuroplasticity, and facilitating functional recovery. These findings highlight the therapeutic potential of BMSCs in treating neurological diseases (Figure 1).

## 3 Research on the related mechanisms of BMSC transplantation in brain neuroplasticity

**FIGURE 2:**
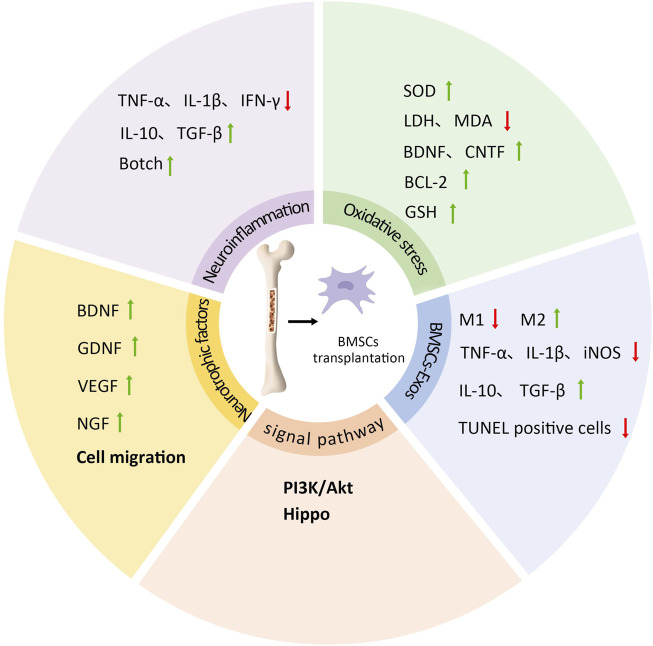
The mechanism of BMSCs transplantation on neural plasticity. BMSCs transplantation can inhibit the expression of pro-inflammatory cytokines TNF-α, IL-1β and IFN-γ, promote the expression of anti-inflammatory cytokines IL-10 and TGF-β, regulate Botch signaling pathway, so as to improve neuroinflammation; BMSCs transplantation increases SOD content, decreased apoptosis, LDH activity and MDA content, and increased the expression of BDNF, CNTF, BCL-2 and GSH. They can regulate oxidative stress response; BMSCs transplantation can also promote the release of neurotrophic factors and cell migration, including the upregulation of BDNF, GDNF, VEGF and NGF; PI3K/Akt and Hippo signaling pathways are also involved; BMSCs-Exos can promote the increase of M2 phenotype and the decrease of M1 phenotype, while IL-1β, TNF-α, iNOS and other cytokines are reduced, IL-10 and TGF-β are increased, and the number of TUNEL positive neurons is reduced, so as to promote neural plasticity and neural recovery.

Research has confirmed the beneficial effects of BMSC transplantation on neuroplasticity, including neurogenesis, neurotrophic factor expression, and synaptic plasticity. Most studies attribute the neuroprotective and reparative benefits of BMSCs on damaged neurons to multiple factors such as transdifferentiation, cell fusion, and growth factor production, while continuously exploring the underlying molecular mechanisms (Zhu et al., 2024). Current understanding primarily involves improvements in neuroinflammation, oxidative stress, cell survival and regeneration, neurotrophic factors, and cell migration processes. Additionally, exosomes secreted by transplanted BMSCs have been shown to influence neuroplasticity (Figure 2). Nevertheless, the potential mechanisms underlying the neuroplasticity benefits of BMSC transplantation require further investigation.

### 3.1 Neuroinflammation and impact of BMSC transplantation on neural plasticity

Inflammation is one of the important influencing factors of brain injury-related diseases. In the central nervous system, microglia and astrocytes are two important cells associated with neuroinflammation. Astrocytes are involved in immune signal regulation (Giovannoni and Quintana, 2020). Microglia are the main immune cells in the central nervous system and play a crucial role in immune regulation and inflammatory responses. Microglia are in a classic balance between pro-inflammatory (M1 phenotype) and anti-inflammatory (M2 phenotype), and their phenotypic changes are related to the local microenvironment (Lee et al., 2016). Microglia can be induced to M1 polarization by lipopolysaccharides (LPS) or interferon gamma and induced to M2 polarization *in vitro* by interleukins IL-4, IL-10, and transforming growth factor alpha (Ślusarczyk et al., 2018). Therefore, the balance of M1/M2 polarization can be regulated to suppress neuroinflammatory responses, which is of great significance for protecting the nervous system from secondary damage (Bok et al., 2018). BMSCs are low immunogenicity pluripotent stem cells with immunosuppressive and anti-inflammatory properties. They can differentiate into neurons, astrocytes, and oligodendrocytes *in vitro* and *in vivo* and mediate inflammation by affecting the activity of microglia or astrocytes, thereby improving the inflammatory state in the brain (Liu GY. et al., 2021).

Transplantation of BMSCs plays a role in promoting neural plasticity by mediating inflammation. In the multiple sclerosis mouse model after BMSCs transplantation, the expression of TNF-α, IL-1 β, and IFN-γ was significantly downregulated, while IL-10 and TGF-β were significantly upregulated, indicating that transplanted BMSCs can alter the expression of inflammatory cytokines (Liu GY. et al., 2021). However, the bone immune suppressive and anti-inflammatory effects of BMSCs require further research. Previous studies reported that JNK signaling is involved in the protective effect of BMSC transplantation, and it can be activated by pro-inflammatory cytokines such as TNF-α, IL-1 β, and various forms of environmental stress. Injecting BMSCs 3 h after MCAO significantly reduced neuronal apoptosis and astrocyte activity in the penumbra, while BMSC transplantation significantly reduced JNK phosphorylation (Vahidinia et al., 2019). Activated microglia can mediate neuroinflammation. Intravenous injection of BMSCs into mice can inhibit the activation of microglia, reduce the expression of inflammatory cytokines, and increase Botch (an endogenous inhibitor of Notch1), thereby inhibiting activation of the Notch1 signaling pathway. When Botch is knocked out in BMSCs, the protective effect of BMSCs completely disappears 24 h after subarachnoid hemorrhage, this finding suggests that the Notch1 signaling pathway may be one of the factors regulating neuroinflammation in BMSC transplantation (Liu et al., 2019). Bai et al. (2021) found that BMSCs can induce CX3CL1 secretion; within 3–7 days after transplantation, the microglia in the infarcted area of MCAO rats were reversed from pro-inflammatory TNF-α to anti-inflammatory CD206 microglia. Therefore, BMSC transplantation can affect neuroinflammation, and vice versa, inflammation may be a factor affecting the proliferation, differentiation, and migration of BMSCs. Studies have also shown that the expression and secretion of inflammatory factors increase under LPS induction, leading to abnormal adipogenic and osteogenic differentiation of BMSCs. However, further research is needed to confirm whether inflammation affects the neuroplasticity of BMSCs after transplantation (Zhou et al., 2021).

Low inflammatory state is believed to be associated with reduced neuroplasticity (Szabó et al., 2014), which can affect neuronal death and neuroplasticity, such as neurogenesis, synapsis, and neurochemical changes in the central nervous system (Ciancarelli et al., 2022). In addition, immune activation and inhibition can impair neural plasticity, such as the expression of LTP and BDNF (Golia et al., 2019). Therefore, inflammation is at least involved in the regulation of neural plasticity. Considering the impact of BMSC transplantation on inflammation and the relationship between inflammation and neuroplasticity, BMSCs affect neuroplasticity by mediating inflammation. However, many questions should be answered in future research, including what triggering factors are involved in the impact of BMSCs on inflammation, is cell differentiation the first to occur, or are changes in signaling pathways related to inflammation?

### 3.2 Oxidative stress and the impact of BMSC transplantation on neural plasticity

Oxidative stress is another important influencing factor of brain injury-related diseases. Mitochondrial dysfunction and oxidative phosphorylation uncoupling mainly occur in the early stages of brain injury, producing many reactive oxygen species (ROS), which lead to widespread oxidative stress, misfolding of proteins in neurons, and ultimately resulting in cell death (MohanKumar et al., 2023). Hence, we believe that oxidative stress is a factor involved in regulating neural plasticity. Recent studies have shown that BMSCs transplantation can regulate the oxidative stress microenvironment through multi-dimensional, and then synergistically promote neural plasticity. In PC12 cells induced by hypoxia, BMSCs increased cell viability and SOD content, while reducing cell apoptosis, LDH activity, and MDA content (Zhou et al., 2019). In retinal ganglion cells, BMSCs downregulated the intracellular oxidative factor MDA, upregulated the intracellular antioxidant factor SOD, and increased the expression of neurotrophic factors BDNF and ciliary neurotrophic factor (CNTF), indicating that BMSCs may exert their protective effect by enhancing antioxidant capacity (Cui et al., 2017). These results suggest that the protective effect of BMSC transplantation may be related to oxidative stress. Calió et al. (2014) found that transplantation of BMSCs can effectively reduce oxidative stress levels in the brain tissue of stroke spontaneously hypertensive (SHRSP) rats, manifested by reduced superoxide and lipid peroxidation in the hippocampus. The increase in level of the anti-apoptotic B-cell lymphoma 2 (Bcl-2) gene and the decrease in Bcl-2 level were closely related to intracellular oxidative stress levels. Intravenous injection of BMSCs increased the levels of five-oxoproline and GSH in MCAO rats as well as the expression of key enzymes involved in GSH synthesis, including gamma glutamyltransferase and gamma glutamylcysteine ligase, indicating that oxidative stress may be influenced by BMSC transplantation (Lan et al., 2022). During the acute phase of neurological disorders, BMSCs may deliver functional mitochondria to neurons through tunneling nanotubes, resulting in increased ATP content and decreased MDA levels (Islam et al., 2012; Li et al., 2014). In the subacute phase, they enhance neurogenesis by upregulating Nrf2 nuclear translocation and subsequent HO-1/SOD2 expression, while potentiating BDNF/TrkB signaling (Kensler et al., 2007). During the chronic phase, BMSCs exert neuroplasticity effects by improving synaptic structure and function. In summary, these results provide evidence for the benefits of BMSCs on neural plasticity through mediating oxidative stress.

To elucidate the mechanism of the protective effects of BMSCs, scholars have studied several signaling pathways related to oxidative stress. The protective effect of BMSCs has been observed in different organs, including the liver, pancreas, brain and bone, and is involved in various diseases. In terms of the role of BMSCs, they have great potential in treating fibrosis. In a rat model of liver fibrosis, administration of BMSCs can increase the expression of Nrf2 and HO-1, providing evidence that BMSCs inhibit oxidative stress by activating the Nrf2/HO-1 signaling mechanism (Khadrawy et al., 2021). In a rat model of severe acute pancreatitis, BMSCs inhibited oxidative stress through Nrf2 nuclear translocation (Zhao et al., 2022). In the brain, BMSCs can alleviate oxidative stress, increase cell viability, and reduce neuronal apoptosis by downregulating the JAK/STAT3 pathway, thereby playing a neuroprotective role in ischemic brain injury (Zhou et al., 2019). Recent studies have revealed that the majority of transplanted BMSCs undergo rapid apoptosis post-transplantation. During this apoptotic process, they generate substantial amounts of apoptotic cell-derived extracellular vesicles (ApoEVs). These vesicles have been demonstrated to facilitate craniofacial bone repair and regeneration through activation of the ROS/JNK signaling pathway (Li et al., 2022).

Overall, BMSC transplantation promotes neural plasticity at least partially by mediating oxidative stress. Some signaling pathways are involved in the protective effects of BMSCs on different tissues and organs. However, whether oxidative stress-related signaling pathways are involved in the regulation of neural plasticity remains unclear. Further research is needed on how to utilize this useful information to improve neural plasticity through BMSC transplantation.

### 3.3 Cell survival and regeneration affecting neural plasticity through BMSC transplantation

#### 3.3.1 PI3K/Akt signaling pathway

The transplantation of BMSCs can regulate cell proliferation, differentiation, migration, and maturation, but its underlying molecular mechanisms are still unclear. Studies have reported on several signaling pathways involved in neurogenesis. The phosphatidylinositol 3-kinase/protein kinase (PI3K/Akt) signaling pathway is involved in regulating various cellular processes, such as cell proliferation, survival, and differentiation. In the rat MCAO model, BMSC transplantation is effective in repairing and rebuilding the brain while reducing the number of apoptotic cells and significantly increasing the expression of P-Akt (Lin et al., 2021). If LY294002 is used to block the PI3K pathway, then the protective effect of BMSCs will be reduced (He et al., 2019). This finding confirms the crucial role of the PI3K/Akt signaling pathway in the benefits of BMSCs.

#### 3.3.2 Hippo signaling pathway

The Hippo pathway is regulates various cellular processes, including cell survival, proliferation, and differentiation. The transplantation of BMSCs can alleviate brain damage caused by cerebral hemorrhage and protect astrocytes from apoptosis. Chen et al. (2020) reported that the transplantation of BMSCs into the brain of mice significantly improved brain injury; it may play a role by regulating the expression of mammalian sterol 20-like kinase (MST) one and Yes-associated protein (YAP) and protecting astrocytes from apoptosis. Given that MST1 is a component of the Hippo pathway and YAP is the main downstream mediator of the Hippo pathway, BMSCs may exert their effects by regulating the Hippo signaling pathway after transplantation. We believe that this process may involve other pathways, but current research data have not been systematically analyzed, so further research is needed to expand the relevant mechanisms.

Although researchers have made great efforts to elucidate the protective mechanism of BMSCs on neural plasticity, many unknown factors exist; when cell proliferation, differentiation, and migration occur after BMSC transplantation; if some intervention measures can be taken, which time is the best; and which indicator can be used for each process? Accurately answering the above questions will provide useful information on the impact of BMSCs on neural plasticity, thereby supporting functional improvement.

### 3.4 Neurotrophic factors, cell migration, and the impact of BMSC transplantation on neural plasticity

#### 3.4.1 Neurotrophic factors

In general, BMSCs are cells that can secrete neurotrophic factors, and their phenotype is similar to that of astrocytes. These cells can secrete a range of neurotrophic factors, such as BDNF, GDND, VEGF, and NGF (Li et al., 2024; Tsai et al., 2021). Thus far, neurotrophic factors secreted by BMSCs promote endogenous neurogenesis, reduce cell apoptosis, and allow neural progenitor cells to migrate to the ischemic site after BMSC transplantation, differentiate into mature neurons, and then survive. However, the mechanism of action of BMSC transplantation in this process is currently unclear.

#### 3.4.2 Cell migration

Cell migration is an important process that must be considered in neurogenesis. To determine which factors that affect cell migration after BMSC transplantation, scholars evaluated the expression of some chemokines and PLY enzymes. Results showed significant increases in chemokines such as monocyte chemoattractant protein-1 (MCP-1), stromal cell-derived factor (SDF)-1α, and polysialytic enzyme ST8SiaIV after BMSC transplantation (Shiota et al., 2018). In addition, CXCR4 is a receptor for SDF-1 α and is involved in the migration of BMSCs in the central nervous system (Tan et al., 2022). The migration of BMSCs is associated with the upregulation of phosphorylated ERK, focal adhesion kinase (FAK), and Akt (Dubon and Park, 2016). In a study aimed at determining the mechanism by which substance P (a neuropeptide) induces the migration of BMSCs, they found that the migration of BMSCs is associated with several classical intracellular signaling pathways, such as the Akt and MAPK/ERK1/2 signaling pathways (Bulj et al., 2013; Gharibi et al., 2012; Rodrigues et al., 2010).

We believe that in the middle stage of neurological disease, the main function is to reshape the neural structure. After BMSCs transplantation, the structural foundation for subsequent functional recovery is laid through the three-level linkage of cell autonomous survival signal, neurotrophic factor and cell migration.

### 3.5 Extracellular vesicles derived from BMSCs exert neuroprotective effects

Extracellular vesicles are nano-sized vesicles with a diameter range of 30–150 nm and possess active biomolecules, such as lipids, proteins, mRNA, and miRNA. These vesicles play an important role in intercellular communication between various cell types and has become a key mediator of stem cell paracrine action. According to reports, extracellular vesicles can cross the blood-brain barrier and target nerve cells because of their nanoscale size. Literature suggests that the protective effect of BMSCs may be attributed to paracrine signaling through secretory exosomes.

Increasing lines of evidence confirms the efficacy of BMSC-derived exosomes (BMSCs-Exos) in antioxidation, anti-inflammatory, angiogenesis, and neurogenesis. Research has shown that BMSCs-Exos can regulate neuroinflammation. Administering BMSCs-Exos to mice 15 min after traumatic brain injury (TBI) can inhibit the expression of pro-inflammatory cytokines TNF-α and IL-1 β. In addition, BMSCs-Exos regulate microglial/macrophage polarization by downregulating iNOS expression and upregulating differentiation cluster 206 and arginase-1 expression (Ni et al., 2019). Consistent with this study, the researchers also confirmed the role of BMSCs-Exos in inflammation regulation; this effect is mainly achieved by regulating the polarization of microglia M1/M2 (Liu X. et al., 2021; Zhang et al., 2022) from pro-inflammatory function to anti-inflammatory function. During this process, the M2 phenotype of microglia increases and the M1 phenotype decreases, while cytokines such as IL-1 β, TNF-α, and iNOS decrease, whereas IL-10 and TGF-β increase, ultimately alleviating inflammation-mediated neurotoxicity. Researchers further found that the TLR/IRK1/NF-κ B signaling pathway is involved in the regulation of inflammation (Zhang et al., 2022).

In addition to inflammation mediation, BMSCs-Exos significantly reduced the levels of pro apoptotic factors, cleaved caspase-3, and cleaved caspase-9 after TBI *in vivo*. BMSCs-Exos significantly reduced the number of TUNEL positive neurons, indicating that BMSCs-Exos can protect neurons from apoptosis after TBI. They found that these changes are related to the p38 MAPK signaling pathway (Zhuang et al., 2022). Co-culture with BMSCs in hypoxia-glucose deprivation/reperfusion-exposed BV-2 cells accelerated cell proliferation, downregulated IL-1β, IL-6, and TNF-α, increased SOD levels, and decreased MDA levels and apoptosis to confirm the protective effects of BMSCs-Exos. Importantly, all of the above effects could be counteracted by inhibition of exosome secretion to counteract. Hence, exosomes play a crucial role in functions, such as anti-inflammation and antioxidation in BMSCs, thereby attenuating apoptosis and accelerating cell proliferation (Yang and Chen, 2022).

BMSCs-Exos can increase neurogenesis and expression of nerve growth factors in MCAO rats, alleviate nuclear vacuolization and demyelination, increase SYN expression in axons and dendrites, and promote myelin remodeling. In addition, BMSCs-Exos can increase the number of axons per neuron and the total axon length. All these data indicate that BMSCs-Exos promote axonal remodeling and enhance axonal growth (Wei et al., 2022). Therefore, BMSCs-Exos have shown great potential in improving neural plasticity. Extracellular vesicles derived from bone marrow stromal stem cells carrying specific cargo, such as protein BDNF, Zeb2/Axin2, or miR146a-5p/124-3p, have shown a significant promoting effect on neuroplasticity (Duan et al., 2020; Min et al., 2022). It also provides a useful method for promoting neural plasticity and recovery under specific conditions, such as ischemic stroke or hemorrhagic brain injury. We believe that in the late stage of neurological disease, the main way is to reconstruct the functional circuit, in which the release of exosomes plays an important role.

## 4 Conclusion

Stem cell transplantation plays a positive role in nerve regeneration and alleviating brain diseases. Research on BMSCs transplantation for the treatment of various diseases has received widespread attention and has been proven to be important as a promising therapy for neurological disorders. BMSC transplantation can have an impact on neural plasticity, including neurogenesis, neurotrophic factors, and synapses. Its various therapeutic mechanisms and effects have been revealed, and it has been proposed as a biological cell therapy for tissue repair and regeneration. Currently, targeting neuroinflammation and oxidative stress may be a promising therapeutic strategy for treating brain injury-related diseases. Furthermore, BMSC transplantation exhibits spatiotemporal dynamic regulation in the treatment of neurological disorders. During the early phase, it primarily ameliorates neuroinflammation and oxidative stress; in the intermediate phase, it mainly enhances cell survival signals, neurotrophic factors, and cell migration; while in the late phase, it promotes the reconstruction of functional circuits through exosome release. These findings significantly promote neural plasticity and provide new ideas and methods for nerve repair and disease treatment. Nonetheless, the mechanisms of how BMSCs transplantation plays a protective role in neuroplasticity are still worth exploring. Understanding how each factor interacts and influences the benefits of BMSCs is critical to developing strategies for BMSCs in the treatment of neurological disorders. In the future, with the development of clinical trials, we believe that stem cells will bring great benefits to patients with neurological disorders.

## Abbreviations

BMSCs, bone marrow mesenchymal stem cells; BDNF, brain-derived neurotrophic factor; bFGF, basic fibroblast growth factor; BMSCs-Exos, bmscs derived exosomes; CNTF, ciliary neurotrophic factor; ERK1/2, extracellular signal-regulated kinase 1/2; FAK, focal adhesion kinase; GDNF, glial cell line-derived neurotrophic factor; GAP43, growth associated protein43; IGF-1, insulin growth factor 1; IL, interleukin; JNK, c-jun n-terminal kinase; MCAO, middle cerebral artery occlusion; MST, mammalian sterile20-like kinase; MAPK, mitogen-activated protein kinase; MCP-1, monocyte chemoattractant protein-1; NPCs, neural progenitor cells; NGF, nerve growth factor; NT-3, neurotrophin-3; PSA-NCAM, polysialylated neural cell adhesion molecule; PI3K/Akt, phosphoinositide 3-kinase/protein kinase; SVZ, subventricular zone; SYP, synaptophysin; SDF, stromal cell derived factor; TBI, traumatic brain injury; VEGF, vascular endothelial growth factor; YAP, yes-associated protein.
